# One-Stage Surgical Outcomes for Bilateral Developmental Dysplasia of the Hip in Walking Children: A Retrospective Case Series Study From the Post-war Era in a Tertiary Care Hospital at Kabul, Afghanistan

**DOI:** 10.7759/cureus.99520

**Published:** 2025-12-18

**Authors:** Sayed Emad, Shekaib R Behroz, Salahuddin Siraj, Mirza Mohammad, Hussain Ali, Rahim Hussain

**Affiliations:** 1 Trauma and Orthopaedics, Kettering General Hospital, Kettering, GBR; 2 Orthopaedics, French Medical Institute for Mothers and Children, Kabul, AFG; 3 Orthopaedic Surgery, French Medical Institute for Mothers and Children, Kabul, AFG; 4 Trauma and Orthopaedic Surgery, Kettering General Hospital, Kettering, GBR; 5 Surgery, South Warwickshire University NHS Foundation Trust, Warwick, GBR

**Keywords:** bilateral ddh, femoral shortening, one-stage surgery, pediatric orthopedics, salter's osteotomy

## Abstract

Purpose

Bilateral surgery for developmental dysplasia of the hip in a single stage represents a significant surgical challenge. The present study, therefore, aimed to evaluate the clinical and radiographic outcomes of one-stage surgical management of bilateral developmental dysplasia of the hip in children after the onset of independent walking.

Materials and methods

This retrospective case series was conducted between 2020 and 2024 at the French Medical Institute for Mothers and Children in Kabul, Afghanistan. The study included 36 patients with a mean age of 32 months (range: 18-54 months), all diagnosed with bilateral developmental dysplasia of the hip, accounting for a total of 72 affected hips. Postoperative functional outcomes were assessed using the modified McKay's scoring system, while radiographic evaluation was performed by measuring the acetabular index. The mean follow-up duration was 9.6 months.

Results

In this study, functional outcomes assessed by the modified McKay's criteria demonstrated excellent results in 26 cases (72%), good results in six cases (16.7%), fair results in three cases (8.3%), and poor results in one case (3%). The mean reduction in the acetabular index between preoperative and postoperative evaluations was 13.78±3.5° for the right hips and 17.29±5° for the left hips. With respect to postoperative complications, the majority of hips (60, 83.3%) showed no adverse effects; however, subluxation occurred in three hips (4.2%), avascular necrosis of the femoral head in four hips (5.5%), and acetabular dysplasia in five hips (7%).

Conclusion

A one-stage open reduction combined with Salter's osteotomy, with femoral shortening when indicated, is recommended as an effective management strategy for bilateral developmental dysplasia of the hip in children aged 1.5-4.5 years.

## Introduction

Developmental dysplasia of the hip refers to the dislocation of the femoral head, which cannot be properly aligned with the acetabulum, along with associated acetabular dysplasia [1]. Its estimated occurrence is approximately 1-3 cases per 1000 live births globally [2]. Developmental dysplasia of the hip may present unilaterally or bilaterally. Although the precise cause remains unclear, exploration of ethnic and genetic factors is considered important [3]. Factors associated with an increased risk include being a firstborn child, breech presentation at birth, female sex, and having first-degree relatives previously treated for developmental dysplasia of the hip [4].

The prognosis of developmental dysplasia of the hip is strongly influenced by early diagnosis, which allows for more effective and less invasive treatment options. However, a considerable number of cases are not diagnosed until after a child has begun walking. The primary treatment goals are to realign and stabilize the joint to encourage the normal development of the hip and reduce the likelihood of future complications [3].

Managing bilateral developmental dysplasia of the hip presents significant challenges, especially when diagnosis occurs after walking age, particularly in regions with limited medical resources and insufficient awareness among families regarding the condition. A single-stage approach involving bilateral open reduction and Salter's osteotomy, with or without femoral shortening, has demonstrated benefits for both patients and parents [5]. This approach reduces the total duration of hospitalization, requires only one application of the hip spica cast, and may reduce overall surgical costs.

Therefore, the present study aimed to evaluate the clinical and radiographic outcomes of one-stage open reduction combined with Salter's osteotomy, with or without femoral shortening, for the treatment of bilateral developmental dysplasia of the hip after the onset of independent walking.

## Materials and methods

This was a retrospective case series study conducted at the French Medical Institute for Mothers and Children in Kabul, Afghanistan, after obtaining approval from the institute's Ethical Review Committee (approval number: 24-FMIC-ER-25), and the data were collected from the available records of 36 patients who remained with bilateral developmental dysplasia of the hip after walking, due to either failure of conservative treatment or late diagnosis without any other associated condition. The indications for surgery were limping in all patients.

Inclusion and exclusion criteria

We included walking-age children with bilateral idiopathic developmental dysplasia of the hip, diagnosed radiographically using the Tönnis classification (grades I-IV). All children underwent a one-stage bilateral open reduction with Salter's innominate osteotomy, with or without femoral shortening, and had no previous treatment. Only those with adequate postoperative follow-up were analyzed. We excluded children with a history of prior hip surgery, neuromuscular disorders (such as muscular dystrophy, cerebral palsy, or arthrogryposis), syndromic conditions, post-infectious or pathologic hip dislocations, or fixed periarticular contractures that prevented reduction.

Based on the Tönnis classification before the operation, the distribution of the participants' hips was as follows: three hips (4%) were categorized as grade I, seven (10%) as grade II, 20 (28%) as grade III, and 42 (58%) as grade IV. Postoperatively, the distribution shifted significantly, with 69 hips (96%) classified as grade 1, three hips (4%) as grade 2, and none as grades 3 or 4.

The Tönnis classification is a radiographic system used to grade the severity of developmental dysplasia of the hip in children. It is based on the position of the capital femoral epiphysis relative to Perkin's line and the superior acetabular rim on anteroposterior pelvic radiographs (Table 1) [6]. 

**Table 1:** The Tönnis classification

Tönnis grade	Description
Grade I	The capital femoral epiphysis lies medial to Perkin's line
Grade II	The capital femoral epiphysis lies lateral to Perkin's line, but below the level of the superior acetabular rim
Grade III	The capital femoral epiphysis lies at the level of the superior acetabular rim
Grade IV	The capital femoral epiphysis lies above the level of the superior acetabular rim

One-stage bilateral open reduction and Salter's osteotomy with or without femoral shortening were performed at the French Medical Institute for Mothers and Children from 2020 to 2024. The age of the participants ranged from 18 to 54 months. The majority, 27 (75%), of the participants were female, and nine (25%) were male. The mean follow-up period was 11 months (range: 13-36 months). The final clinical assessment of patients was performed according to the modified MacKay's criteria.

The modified McKay's criteria is a clinical grading system used to evaluate functional outcomes in patients with developmental dysplasia of the hip. It assesses key parameters, including pain, hip stability, gait (presence of a limp or Trendelenburg sign), and range of motion of the affected hip. Based on these clinical factors, outcomes are classified into four grades: excellent, good, fair, or poor. This classification (originally described by McKay and later refined by Berkeley et al. in 1984) [7] is widely used in pediatric orthopedics to describe postoperative hip function. The grading categories and their clinical descriptors are shown in Table 2.

**Table 2:** The modified McKay's criteria ROM: range of motion

Grade	Rating	Description
I	Excellent	Stable painless hip, no limp, negative Trendelenburg sign, full ROM
II	Good	Stable painless hip, slight limp, negative Trendelenburg sign, slight decrease in ROM
III	Fair	Stable painless hip, limp positive, positive Trendelenburg sign, limited ROM
IV	Poor	Unstable or painful hip or both, positive Trendelenburg sign

The statistical analysis was performed using IBM SPSS Statistics for Windows, Version 23.0, developed by IBM Corporation in Armonk, New York, United States. As the acetabular index is a continuous variable, a paired t-test was used to compare preoperative and postoperative values. Statistical significance was set at p<0.05 with a 95% confidence interval. A probability-based simple random sampling method was utilized.

## Results

The analysis encompassed 36 sets of medical records (72 hips in total) from pediatric orthopedic patients diagnosed with bilateral developmental dysplasia of the hip and treated at the French Medical Institute for Mothers and Children between 2020 and 2024. Among these, 34 hips underwent one-stage open reduction combined with Salter's osteotomy and femoral shortening, whereas 38 underwent one-stage open reduction along with Salter's osteotomy (Table 3).

**Table 3:** AI values pre- and post-operation (degrees) in relation to the affected side and types of procedure AI: acetabular index; OR, SO: open reduction and Salter's osteotomy; OR, SO, FS: open reduction, Salter's osteotomy, and femur shortening

Patient	Age (months)/sex	Preoperative AI (right)	Preoperative AI (left)	Postoperative AI (right)	Postoperative AI (left)	Type of procedure (right)	Type of procedure (left)
1	18M	30	30	18	19	OR, SO, FS	OR, SO, FS
2	20F	30	50	20	20	OR, SO	OR, SO, FS
3	22F	35	45	19	20	OR, SO	OR, SO, FS
4	24M	38	40	20	20	OR, SO, FS	OR, SO, FS
5	25F	34	34	21	20	OR, SO, FS	OR, SO, FS
6	27M	30	35	19	18	OR, SO, FS	OR, SO, FS
7	28M	33	34	20	19	OR, SO, FS	OR, SO, FS
8	30F	30	30	18	19	OR, SO, FS	OR, SO, FS
9	31F	30	40	20	20	OR, SO, FS	OR, SO, FS
10	33M	30	30	21	20	OR, SO	OR, SO
11	34F	38	48	20	21	OR, SO	OR, SO
12	35F	30	35	19	20	OR, SO	OR, SO
13	36M	33	33	18	21	OR, SO	OR, SO
14	37F	40	42	21	22	OR, SO	OR, SO
15	38M	30	35	18	19	OR, SO	OR, SO
16	39M	28	40	16	20	OR, SO	OR, SO
17	40F	35	40	19	18	OR, SO	OR, SO
18	41F	42	40	20	20	OR, SO	OR, SO
19	42F	40	40	21	20	OR, SO	OR, SO
20	43F	32	30	18	20	OR, SO	OR, SO
21	44M	30	40	20	19	OR, SO	OR, SO
22	45F	32	40	21	22	OR, SO, FS	OR, SO, FS
23	46F	35	40	19	23	OR, SO, FS	OR, SO, FS
24	47M	38	42	20	22	OR, SO, FS	OR, SO, FS
25	48F	30	35	18	20	OR, SO, FS	OR, SO, FS
26	49F	33	34	20	19	OR, SO, FS	OR, SO, FS
27	50F	30	30	19	19	OR, SO	OR, SO
28	51F	33	33	21	20	OR, SO	OR, SO
29	52F	40	42	22	22	OR, SO	OR, SO
30	53F	30	35	19	19	OR, SO	OR, SO
31	54F	40	40	20	20	OR, SO	OR, SO
32	19M	32	30	20	20	OR, SO	OR, SO
33	21F	30	40	21	21	OR, SO, FS	OR, SO, FS
34	23F	30	30	19	19	OR, SO, FS	OR, SO, FS
35	26M	38	48	20	22	OR, SO, FS	OR, SO, FS
36	29F	30	35	18	20	OR, SO, FS	OR, SO, FS

The ages of the participants ranged from 18 to 54 months, with an average age of 32 months. Among the participants, 27 (75%) were females, and nine (25%) were males. Follow-up after the surgery ranged from eight to 24 months. During the study, we observed excellent outcomes in 26 (72%) cases, good outcomes in six (16.7%) cases, fair outcomes in three (8.3%) cases, and poor outcomes in one (3%) case according to the modified McKay's criteria.

The majority of patients (60 hips, 83.3%) encountered no procedure-related complications. However, three hips (4.2%) experienced subluxation, four hips (5.5%) suffered from avascular necrosis (AVN) of the femoral head, and five hips (7%) showed signs of acetabular dysplasia.

Furthermore, there was a statistically significant difference in the mean acetabular index between preoperative and postoperative measurements. Specifically, the mean difference was 13.778±3.5 for the right side and 17.287±5 for the left side. This significance was confirmed by a p-value of <0.001.

The acetabular index is the angle formed between Hilgenreiner's line and a line drawn along the acetabular roof on an anteroposterior pelvic radiograph. It reflects acetabular development, with normal values averaging ~27° at birth, <25° by one year, and ~20° by two years. An elevated acetabular index indicates acetabular dysplasia and is widely used to assess developmental dysplasia of the hip [8]. 

The postoperative acetabular index is used for radiographic outcomes.

Table 4 demonstrates a marked reduction in the acetabular index of right-sided hips postoperatively. The mean acetabular index decreased significantly from 33.31°±3.96 preoperatively to 19.53°±1.25 postoperatively, indicating a substantial improvement in acetabular alignment. A paired t-test confirmed that this difference was statistically significant (p<0.001). The variability of acetabular index values also narrowed post-surgery, reflecting more consistent acetabular correction.

**Table 4:** Post-surgical improvement of the AI in the right hip *Significant p-value <0.05 P-values were calculated using a paired t-test. AI: acetabular index

Statistics	Preoperative AI	Postoperative AI	P-value	t-statistic
Minimum	28°	16°	<0.001*	11.42
Maximum	42°	22°	<0.001*	11.42
Mean (±SD)	33.31° (3.96)	19.53° (1.25)	<0.001*	11.42

Table 5 similarly shows a significant reduction in the acetabular index of left-sided hips after surgery. The mean acetabular index decreased from 37.36°±5.53 preoperatively to 20.08°±1.18 postoperatively, with the range narrowing from 30-50° to 18-23°. A paired t-test demonstrated that the difference was highly significant (p<0.001), confirming substantial post-surgical improvement in acetabular alignment.

**Table 5:** Post-surgical improvement of the AI in the left hip *Significant p-value <0.05 P-values were calculated using a paired t-test. AI: acetabular index

Statistics	Preoperative AI	Postoperative AI	P-value	t-statistic
Minimum	30°	18°	<0.001*	12.05
Maximum	50°	23°	<0.001*	12.05
Mean (±SD)	37.36° (5.53)	20.08° (1.18)	<0.001*	12.05

Surgical technique

The operating surgeon thoroughly explained the entire procedure to the parents, who understood the procedure and provided written consent. The procedure was performed under general anesthesia and caudal block, with intravenous cefazolin antibiotics administered immediately before the operation.

The surgeries were conducted with the patient lying on their back using either one or two incisions on each side. For patients requiring only open reduction and Salter's osteotomy, a single-incision approach via the anterior iliofemoral approach (Smith-Petersen) was employed. The spaces between specific muscles were identified and incised. A T-shaped cut was made in the capsule, followed by a transverse incision along the femoral neck. Capsulorrhaphy was performed in all cases.

**Figure 1:**
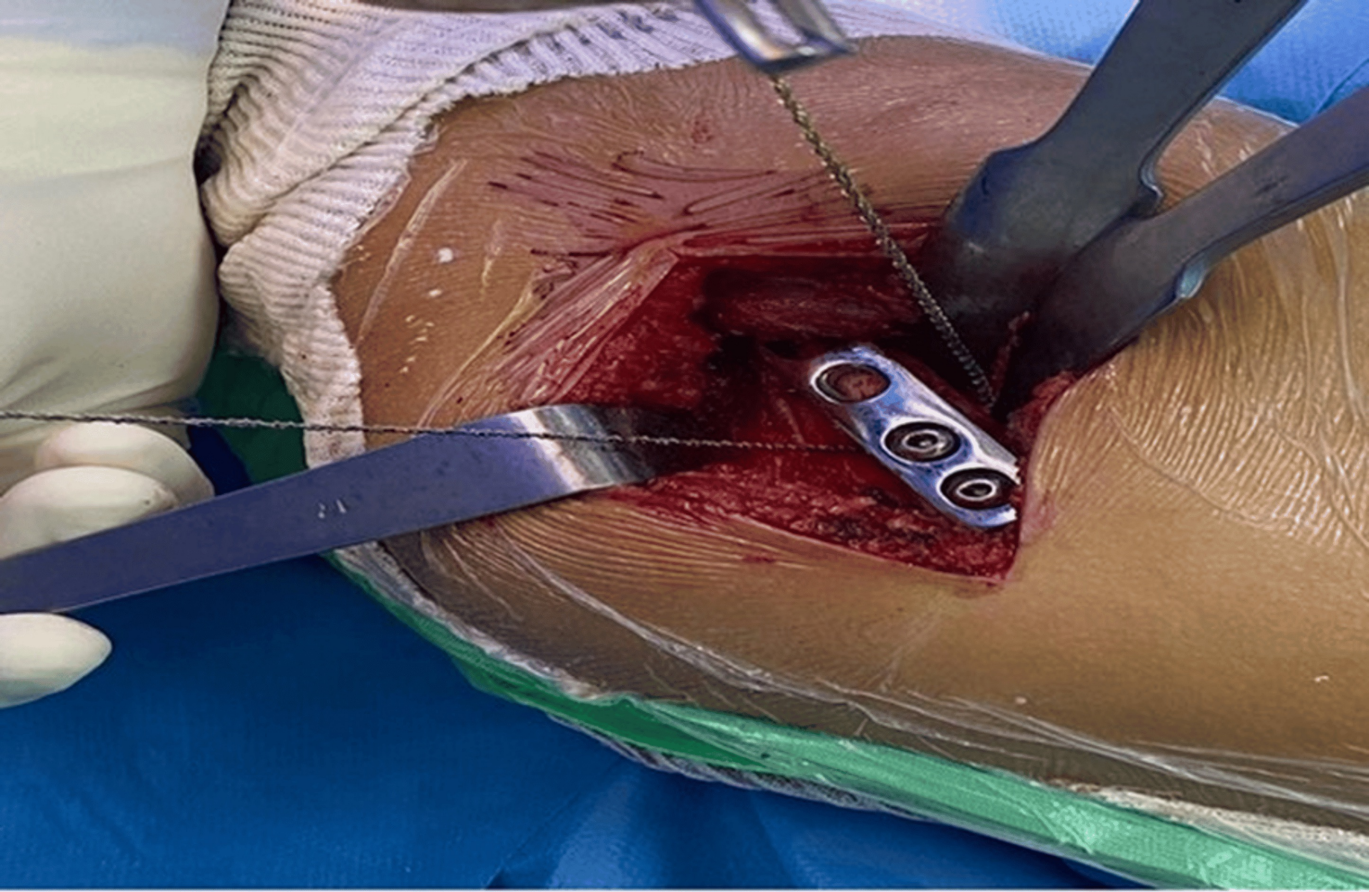
Femoral shortening with the Gigli saw

For patients needing open reduction, Salter's osteotomy, and femur shortening (Figure 1), a two-incision approach was utilized. Femur shortening involved exposing the proximal femur laterally, performing osteotomy with a Gigli saw, and securing it with four- or five-hole plates.

**Figure 2:**
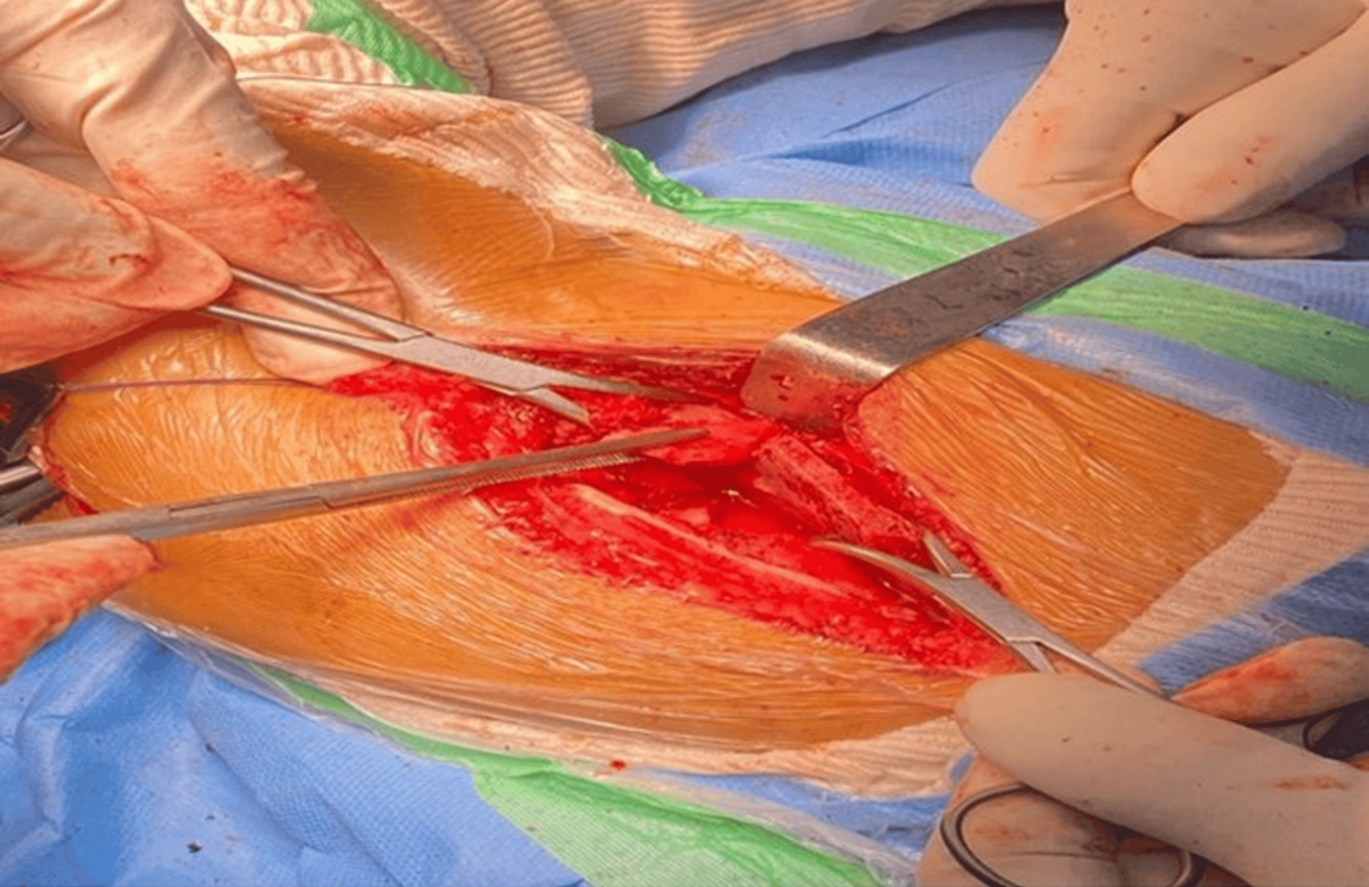
Salter's osteotomy of the hip

Iliac bone osteotomy was performed using a Gigli saw (Figure 2), and the site was opened anterolaterally by repositioning the lower and outer portions of the innominate bone.

**Figure 3:**
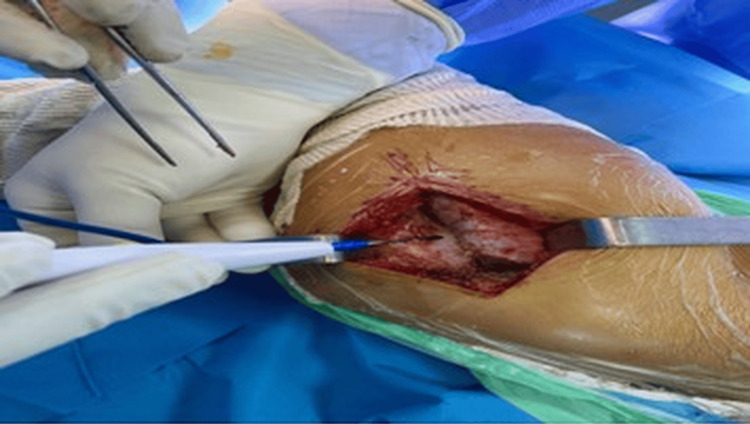
Post-Salter's osteotomy of the affected hip

A full-thickness bone graft from the middle of the iliac bone was placed (Figure 3) and secured using two Kirschner wires at the osteotomy site.

The patients were immobilized with a hip spica cast for two months. Only two patients received a blood transfusion. Intravenous prophylactic cefazolin was administered during the operation and every six hours postoperatively, lasting a total of one day for all patients. Kirschner wires were removed under general anesthesia 8-16 weeks after surgery, and the plates were removed 8-12 months after surgery.

Based on the Tönnis classification before the operation, the distribution of the participants' hips was as follows: three hips (4%) were categorized as grade I, seven (10%) as grade II, 20 (28%) as grade III, and 42 (58%) as grade IV. Postoperatively, the distribution shifted significantly, with 69 hips (96%) classified as grade 1, three hips (4%) as grade 2, and none as grades 3 or 4.

**Figure 4:**
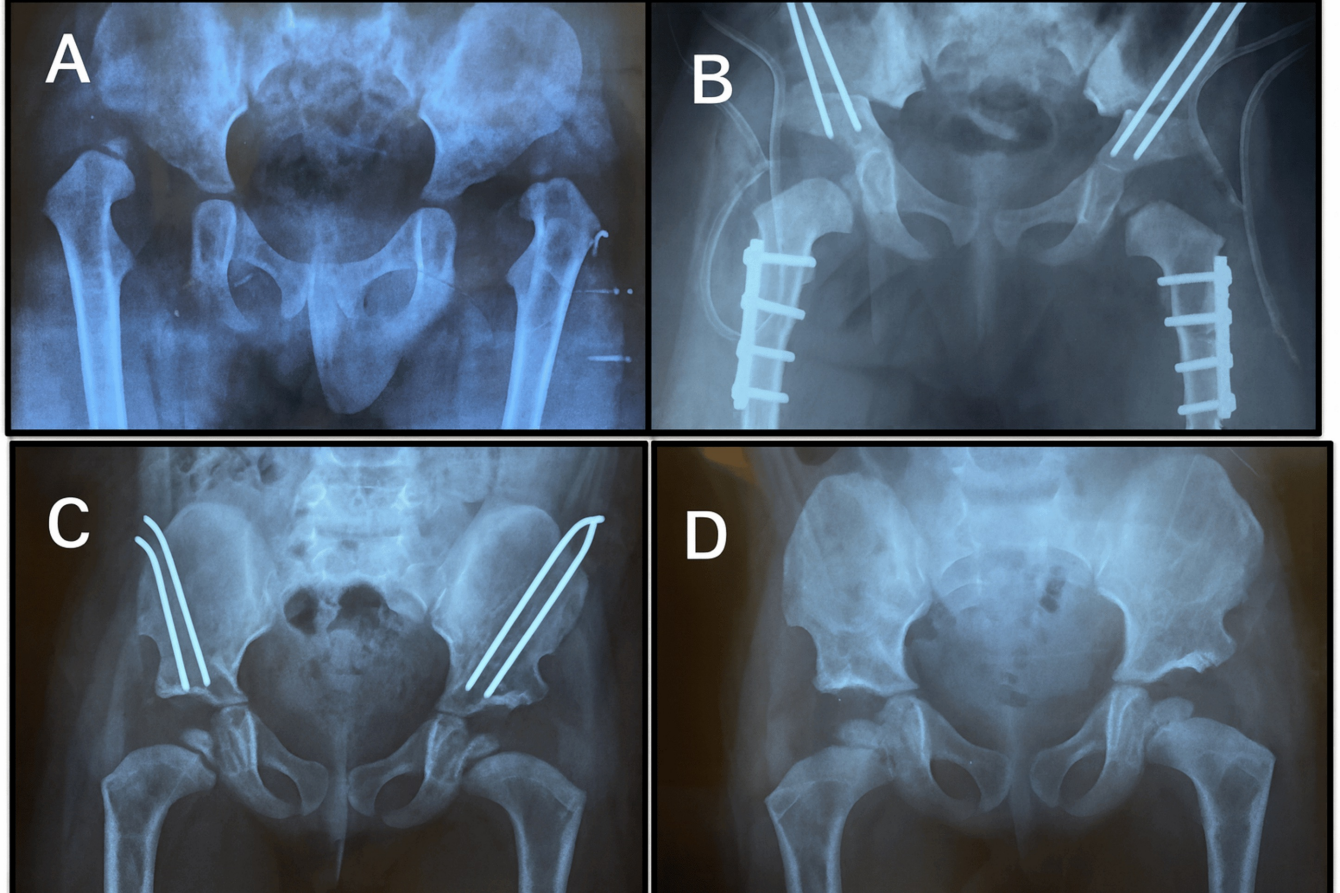
Radiographic progression in a 41-month-old female patient: (A) preoperative appearance, (B) immediate postoperative result, (C) follow-up at 10 months post-operation, and (D) follow-up at 14 months post-operation

Figure 4 shows the radiographic progression in a 41-month-old female patient.

**Figure 5:**
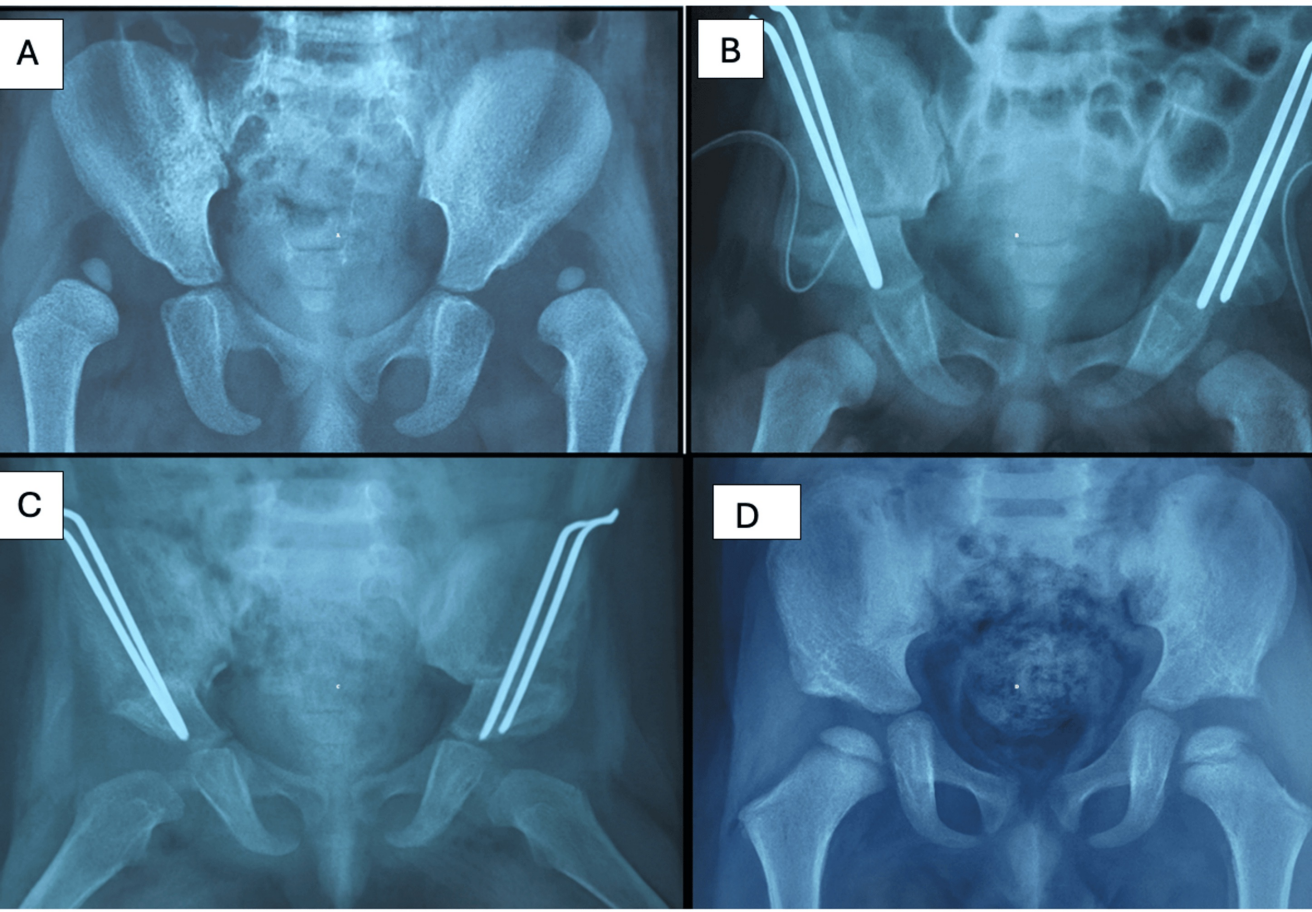
Radiographic progression in a 24-month-old female patient: (A) preoperative appearance, (B) immediate postoperative result, (C) Follow-up at two months after surgery, and (D) follow-up at 18 months after surgery

Figure 5 shows the radiographic progression in a 24-month-old female patient. 

## Discussion

Our one-stage bilateral open reductions with Salter's osteotomy yielded excellent or good clinical outcomes in the vast majority of hips (88.7%). This result is in line with other series of single-stage bilateral developmental dysplasia of the hip surgery. Gem et al. reported 71.8% excellent and 28.2% good results (100% satisfactory) using a similar combined approach [5]. Likewise, Qadir et al. found ~86% of hips had good/excellent McKay outcomes following one-stage reconstruction in older children [9]. These high success rates support the efficacy of comprehensive single-stage correction. Aljanabi and Hakim noted that patients undergoing open reduction alone had marginally better functional scores than those receiving additional osteotomies [10], but both groups showed generally favorable results. Overall, the excellent/good rate in our cohort compares favorably with published data, suggesting that one-stage bilateral management can achieve equally positive functional outcomes as staged or unilateral approaches.

In our series, the mean acetabular index fell dramatically postoperatively (p<0.001), and Tönnis grades improved in all hips, indicating successful concentric reductions. These radiographic improvements mirror those reported after Salter's osteotomy. Aldhoon et al. observed an average acetabular index reduction from 34° to ~20° after Salter's innominate osteotomy [11]. Similarly, Kotzias Neto et al. found significant increases in center-edge angle and decreases in acetabular index following open reduction with Salter's osteotomy, regardless of whether femoral shortening was added [12]. In our patients, the consistency of acetabular index improvement reflects this expected acetabular reorientation.

Salter's innominate osteotomy achieves these radiographic gains by hinging the innominate bone at the pubic symphysis and rotating the acetabular fragment anterolaterally [13]. This maneuver increases lateral coverage and is held in place with a bone graft and fixation [13]. In our hands, the post-osteotomy acetabular index and center-edge angles approached normal values [6], as in other reports [11,12]. In short, the radiographic data strongly suggest that the osteotomy component of our procedure restored acetabular coverage very effectively.

Overall, our complications were relatively infrequent. Per the narrative review by Badrinath et al., the main postoperative concerns in this age group are residual dysplasia, redislocation/subluxation, and osteonecrosis [14]. We saw only 4.2% of hips with subluxation and 7% with residual acetabular dysplasia requiring later surgery, rates well below those reported elsewhere. For example, Gem et al. noted that 22 of 92 hips (23.9%) required a secondary Salter's osteotomy for persistent dysplasia [5]. Similarly, our rate of secondary procedures was markedly lower (7%). AVN occurred in 5.5% of hips in our cohort. By comparison, Zamzam et al. recently reported AVN in 12% of hips and found it to be a strong predictor of poor outcome [15], and Aljanabi and Hakim observed AVN in 34.8% of treated hips (mostly mild, grade I) [10]. Castañeda et al. similarly found that performing femoral shortening did not significantly alter AVN rates [13]. Thus, our AVN rate was low relative to many series. In sum, our profile of a few redislocations, modest reoperation rate, and limited AVN is favorable compared to published data [5,13,14].

All operations in this series were performed in a single session on both hips. Li et al. have shown that sequential bilateral procedures can lead to asymmetric outcomes in a majority of patients (~69%) [16], a problem inherently avoided by true one-stage surgery. Studies directly comparing simultaneous versus staged bilateral developmental dysplasia of the hip surgery have found no significant differences in radiographic or AVN outcomes. Köse et al. reported essentially identical final acetabular indices and AVN rates between simultaneous and delayed bilateral osteotomy groups [17]. In their series, neither approach conferred a clear advantage, suggesting that one-stage correction is safe and effective when expertise allows. Our uniformly positive results with a single anesthesia and hospital stay align with these findings and support the idea that experienced centers may safely perform bilateral open reductions and osteotomies at once [17,18].

Limitations of our study include its retrospective design, relatively small sample size, and mid-term follow-up. We did not systematically capture patient-reported outcomes or long-term arthritic changes. Conversely, a strength is the focus on one-stage bilateral cases in a homogeneous age group (mean: 32 months), which is seldom reported in detail. In summary, our cohort demonstrated high rates of excellent functional outcomes, significant radiographic correction (with improved acetabular index and Tönnis grade), and relatively low complication rates. These results are largely in agreement with recent literature and underscore the viability of one-stage bilateral open reduction plus osteotomy in appropriately selected children.

## Conclusions

This study indicates that a single-stage bilateral open reduction combined with Salter's osteotomy can effectively restore hip stability and functional alignment in children presenting after walking age. This approach allows the simultaneous correction of both hips while promoting improved joint development and balanced mobility.

The procedure demonstrated acceptable safety and minimized the need for repeated hospital admissions, extended anesthesia exposure, and multiple casting periods, thereby reducing the overall treatment burden on families. Although further long-term and larger-scale studies are needed, these findings support the use of this technique as a practical and reliable option for managing late-presenting developmental dysplasia of the hip.
